# External validation of multimodal termination of resuscitation rules for out-of-hospital cardiac arrest patients in the COVID-19 era

**DOI:** 10.1186/s13049-021-00834-0

**Published:** 2021-01-27

**Authors:** Haewon Jung, Mi Jin Lee, Jae Wan Cho, Sang Hun Lee, Suk Hee Lee, You Ho Mun, Han-sol Chung, Yang Hun Kim, Gyun Moo Kim, Sin-youl Park, Jae Cheon Jeon, Changho Kim, on behalf of the WinCOVID-19 consortium

**Affiliations:** 1 Department of Emergency Medicine, School of Medicine, Kyungpook National University, 680, Gukchaebosang-ro, Jung-gu, Daegu, 41944 Republic of Korea; 2 Department of Emergency Medicine, Keimyung University Dongsan Hospital, Daegu, Republic of Korea; 3 Department of Emergency Medicine, Catholic University of Daegu School of Medicine, Daegu, Republic of Korea; 4 Department of Emergency Medicine, Yeungnam University College of Medicine, Daegu, Republic of Korea; 5 Department of Emergency Medicine, Kyungpook National University Chilgok Hospital, Daegu, Republic of Korea; 6 Department of Emergency Medicine, Daegu Fatima Hospital, Daegu, Republic of Korea

**Keywords:** Heart arrest, Prognosis, Coronavirus disease, Ethics, Cardiopulmonary resuscitation

## Abstract

**Background:**

Futile resuscitation for out-of-hospital cardiac arrest (OHCA) patients in the coronavirus disease (COVID)-19 era can lead to risk of disease transmission and unnecessary transport. Various existing basic or advanced life support (BLS or ALS, respectively) rules for the termination of resuscitation (TOR) have been derived and validated in North America and Asian countries. This study aimed to evaluate the external validation of these rules in predicting the survival outcomes of OHCA patients in the COVID-19 era.

**Methods:**

This was a multicenter observational study using the WinCOVID-19 Daegu registry data collected during February 18–March 31, 2020. The subjects were patients who showed cardiac arrest of presumed cardiac etiology. The outcomes of each rule were compared to the actual patient survival outcomes. The sensitivity, specificity, false positive value (FPV), and positive predictive value (PPV) of each TOR rule were evaluated.

**Results:**

In total, 170 of the 184 OHCA patients were eligible and evaluated. TOR was recommended for 122 patients based on the international basic life support termination of resuscitation (BLS-TOR) rule, which showed 85% specificity, 74% sensitivity, 0.8% FPV, and 99% PPV for predicting unfavorable survival outcomes. When the traditional BLS-TOR rules and KoCARC TOR rule II were applied to our registry, one patient met the TOR criteria but survived at hospital discharge. With regard to the FPV (upper limit of 95% confidence interval < 5%), specificity (100%), and PPV (> 99%) criteria, only the KoCARC TOR rule I, which included a combination of three factors including not being witnessed by emergency medical technicians, presenting with an asystole at the scene, and not experiencing prehospital shock delivery or return of spontaneous circulation, was found to be superior to all other TOR rules.

**Conclusion:**

Among the previous nine BLS and ALS TOR rules, KoCARC TOR rule I was most suitable for predicting poor survival outcomes and showed improved diagnostic performance. Further research on variations in resources and treatment protocols among facilities, regions, and cultures will be useful in determining the feasibility of TOR rules for COVID-19 patients worldwide.

**Supplementary Information:**

The online version contains supplementary material available at 10.1186/s13049-021-00834-0.

## Background

The novel coronavirus disease (COVID-19) continues to be in a pandemic status and remains an important health concern worldwide [1]. Due to the presenting symptoms, complications, and transmission efficiency of COVID-19, we changed our standard treatment processes and personal protection strategies. In particular, resuscitation of cardiac arrest patients posed an especially high risk of disease transmission to healthcare professionals [2, 3]. For in-hospital cardiac arrest (IHCA) patients, resuscitation was typically performed after establishing whether the patient has COVID-19. However, for out-of-hospital cardiac arrest (OHCA) patients, resuscitation was performed prior to any testing or confirmation. Consequently, the OHCA group was of high risk to rescuers.

In the prehospital setting, emergency medical personnel screen patients for COVID-19 based on their symptoms (fever, dyspnea, cough, and sore throat) and inquired about any recent history of contact with infected individuals. However, this method has limitations because many carriers are asymptomatic [4, 5]. In addition, these asymptomatic COVID-19 patients admitted to the intensive care unit (ICU) pose a challenge to determining the need for wearing personal protective equipment (PPE) and utilizing medical resources. Patients who failed to experience a return of spontaneous circulation (ROSC) in the event of a cardiac arrest were also often no longer transported to the hospital due to their low survival probability [6]. Accordingly, recent cardiopulmonary resuscitation (CPR) guidelines were changed to recommend that all healthcare providers assess the safety of the scene and wear higher levels of PPE for protection against infectious airborne droplets and detailing the criteria for the basic life support termination of resuscitation (BLS-TOR) rule. These guidelines stipulate that before terminating resuscitative efforts, the healthcare workers must check that (1) the arrest was not witnessed by emergency medical service (EMS) personnel or a first responder, (2) ROSC was not observed prior to the patient being transported to the emergency medical center (EMC), and (3) no electrical shocks were delivered [7–14].

We applied the rules that were typically selected depending on the country or region where the derivation and validation phases were conducted. These included the (1) International BLS and advanced life support (ALS) rules derived and validated in the United States and Europe [8]; (2) Goto and KANTO-SOS rules developed in Japan and Asian countries [13, 14]; and (3) Korean OHCA registry-based TOR models, Korean Cardiac Arrest Research Consortium (KoCARC) TOR rules, and two new TOR models that were used in our previous studies [7, 11].

In Daegu metropolitan city, the number of patients with COVID-19 increased rapidly at the ​​community level from February 18, 2020 concurrent with the pandemic outbreak in South Korea. During its 7-week peak, we resuscitated 184 OHCA patients at six EMCs. This was the first study to determine the efficacy of these TOR guidelines for an emerging infectious disease [7, 11, 12].

We were collecting prospective data for a registry of OHCA patients since 2017. However, as emergency rooms were shutdown, we recognized a need for additional research to improve the outcomes of OHCA patients while concurrently protecting emergency department (ED) staff. Thus, this registry-based observational study aimed to evaluate the external validation of these rules in predicting the survival outcomes of OHCA patients in the COVID-19 era.

## Methods

### Study design and variables

This study was approved by the Institutional Review Board of Kyungpook National University Hospital (Approval no.: KNUH 2020–04-032).

We conducted a multicenter registry-based observational study using the WinCOVID-19 citywide OHCA registry data. In March 10, ED personnel from six hospitals in Daegu voluntarily discussed about data of COVID-related factors. We evaluated patient demographics (age and sex), prehospital variables (e.g., place of arrest, initial cardiac rhythm, whether the arrest was witnessed by a bystander or EMS personnel, whether a bystander performed CPR, whether an automated external defibrillator was used and prehospital defibrillation occurred, and whether ROSC occurred), and time intervals. Response time was defined as the period from contacting the EMS to them arriving at the scene, while scene time was defined as the period from the EMS arriving at the scene to them departing from the scene. Resuscitation management and survival outcomes, including ROSC, survival to admission, survival to discharge, and neurological outcomes at discharge, after cardiac arrest were recorded as hospital-level variables. Based on this collective data, we determined whether the patients met the existing TOR guidelines and qualified to be enrolled in this study. In South Korea, EMS personnel cannot terminate resuscitation unless OHCA patients show obvious signs of death [7, 11]; therefore, all EMS-treated OHCA patients should be transported to the hospital. Notably, these personnel use manual defibrillators and analyze electrocardiograms prior to the patients’ arrival at the hospital.

To evaluate COVID-related variables, we obtained data, including the clinical symptoms or signs (sore throat and cough), vital signs (fever was defined as a temperature of ≥37.5 °C]), recent exposure history, and chest X-ray findings, of patients with confirmed COVID-19 on admission from the electronic medical records.

### Study setting and patients

Daegu is a metropolitan city with a population of 2.44 million people and is composed of two regional level I EMCs, four local level II EMCs, and 19 emergency facilities or clinics. In particular, there are six EMCs where ALS, post-cardiac arrest care, and cardiovascular interventions are available. In this study, we evaluated adult patients (age > 18 years) who presented with presumed OHCA and used the EMS system in Daegu. Those who did not receive any resuscitation and those who experienced cardiac arrest in a primary care clinic or long-term care hospital were excluded.

### Main outcome measures

The primary outcome measures were (1) the proportions of survival to hospital discharge defined as discharged to go home or transferred to another facility after admission to the hospital and (2) survival with a favorable neurological outcome quantified using Cerebral Performance Category scores, with scores of 1 and 2 being the most favorable.

Meanwhile, the secondary outcome measures included external validation of previously researched TOR rules (Table 1). We identified OHCA patients who met all the TOR criteria and calculated the sensitivity, specificity, false positive ratios (FPR), false positive values (FPVs), positive predictive values (PPVs), negative predictive values (NPVs), and their respective 95% confidence intervals (CI) to identify patients with a risk of poor survival [7, 8, 11, 13, 14].

**Table 1:** Criteria of the BLS-TOR and KoCARC BLS-TOR rules

TOR rules	Witness status	Initial prehospital rhythm	Prehospital shock	Prehospital ROSC	Others
**Based on prehospital criteria**					
International BLS rule^8^	not witnessed by EMT		no prehospital shock	no prehospital ROSC	
International ALS rule^8^	not witnessed by bystander/EMT		no prehospital shock	no prehospital ROSC	no bystander CPR
Goto’s TOR rule^13^	not witnessed by bystander	Initial non-shockable rhythm		no prehospital ROSC	
KoCARC TOR rule I ^7^	not witnessed by EMT	asystole in prehospital conditions	no prehospital shock	no prehospital ROSC	
KoCARC TOR rule II	not witnessed by EMT		no prehospital shock	no prehospital ROSC	Age > 60
KoCARC TOR rule III	not witnessed by EMT	asystole in prehospital conditions	no prehospital shock	no prehospital ROSC	Age > 60
New TOR model 1^11^	not witnessed by bystander	asystole in the field		no prehospital ROSC	
**Based on prehospital and ED**					
SOS-KANTO’s TOR rule^14^	not witnessed by bystander	asystole in the field			asystole in the hospital
New TOR model 2	not witnessed by bystander			no prehospital ROSC	asystole in the hospital

*ALS* advanced life support, *BLS-TOR* basic life support and termination of resuscitation, *CPR* cardiopulmonary resuscitation, *EMT* emergency medical technicians, *KoCARC* Korean Cardiac Arrest Research Consortium, *ROSC* return of spontaneous circulation, *SOS-KANTO* survey of survivors after cardiac arrest conducted in the Kanto area in 2012 (2017)

### Statistical analyses

The normality of the variables was determined using the Shapiro–Wilk test. Descriptive statistics were presented as medians with interquartile ranges (IQR, 25th and 75th percentiles), whereas categorical variables were presented as amounts and percentages. Significant differences among the survival outcomes were determined using the Mann-Whitney test for continuous variables and chi-square tests or Fisher’s exact test for categorical variables.

Logistic regressions were performed for patients who met the criteria of the previous TOR rules. The outcome of interest was survival to hospital discharge (Table 1). To determine the model calibration, the Hosmer-Lemeshow goodness of fit test was calculated. Odds ratio (OR) greater than 1 indicated an unfavorable effect on survival; ORs and 95% CIs were calculated for all covariates. The characteristics of the adjusted ORs were described using Forest plots. A receiver operating characteristic (ROC) curve, with the area under the curve (AUC), was used to determine the accuracy of such variables in predicting unfavorable survival outcomes at discharge. All tests were two-tailed, and *p* < 0.05 was considered statistically significant.

The diagnostic performances of all existing TOR rules were analyzed. Previous TOR rules were created based on FPR (1-specificity, i.e., the probability that the rule will suggest terminating resuscitation in case of patient survival) and PPV (i.e., the probability that the rule will suggest terminating resuscitation in case of patient death) of the rules. Current guidelines recommend that when cessation of life-sustaining care is considered, the tool used to predict poor outcomes must be accurate and reliable, with an specificity (of close to 100%) and a PPV (of > 99%, a narrow 95% CI) [7, 15].

All statistical analyses were performed using IBM SPSS Statistics v. 25 (IBM Corp., Armonk, NY, USA) and MedCalc v. 17.4.4 (MedCalc Software, Mariakerke, Belgium).

## Results

### Patient characteristics

**Fig. 1:**
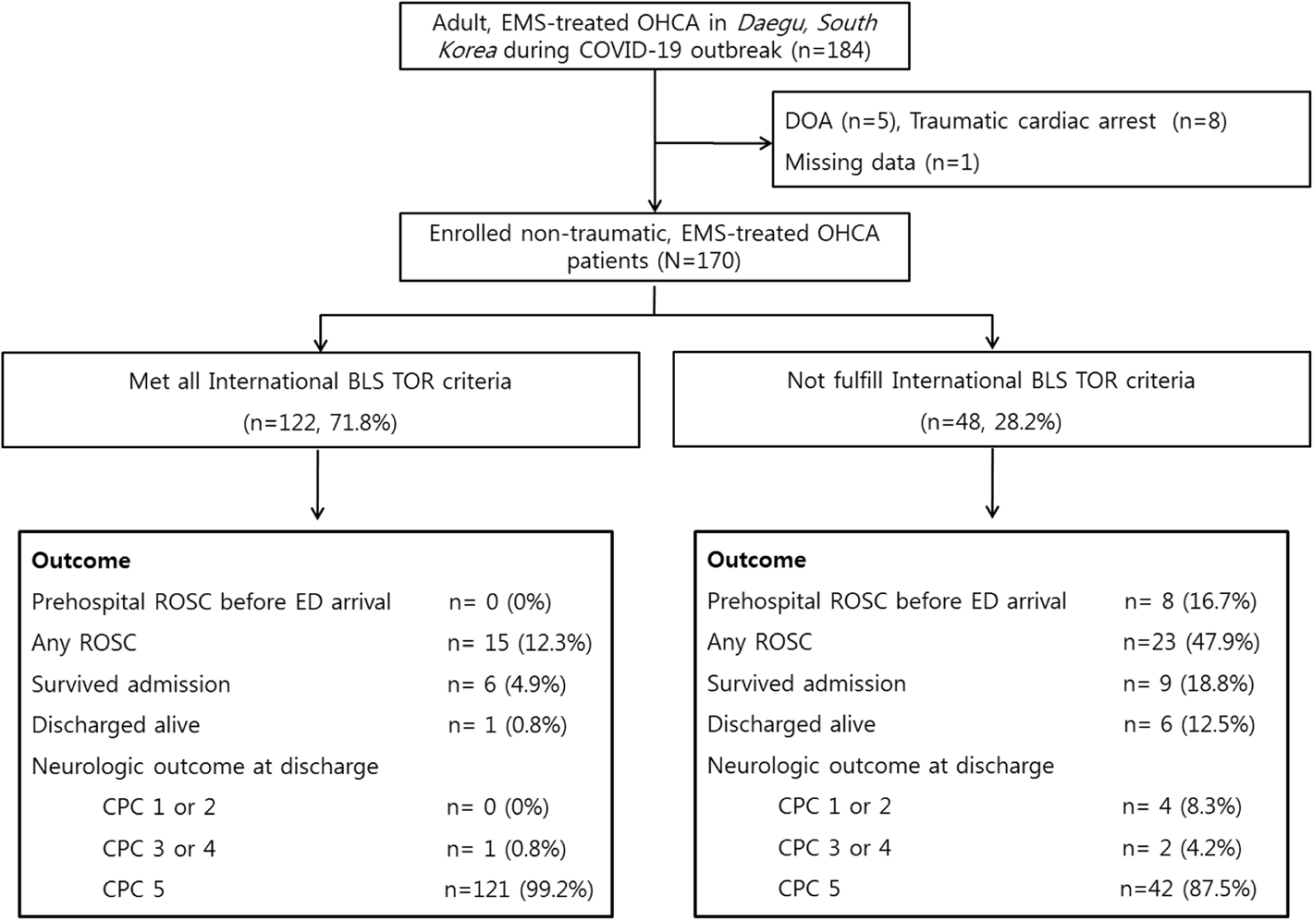
Study flowchart

Demographic and prehospital characteristics of the 2017–2018 Korean nationwide OHCA and Daegu Citywide OHCAs were presented in Supplementary Table 1. The prehospital and hospital characteristics of variables are almost similar. Of the 184 adults who developed OHCA between February 18, 2020 and March 31, 2020 (i.e., the peak of the COVID-19 outbreak), 170 patients (male, 63.2%) were included in this study (Fig. 1). In total, 76.0% of the arrest was witnessed by EMS personnel, 34.3% of the patients received CPR from a bystander, 4.7% experienced ROSC prior to hospital arrival, 58.5% showed abnormal chest radiography findings, and 5.8% were confirmed to have COVID-19 via reverse transcription polymerase chain reaction. The patients’ baseline characteristics are summarized in Table 2.

**Table 2:** General characteristics of the COVID-19 OHCA study population

	Overall (***n*** = 170)	Survival at discharge (***n*** = 7)	Dead (***n*** = 163)	***P*** value
**Age (years), median [IQR]**	74 [62–80]	62 [57–69]	75 [63–80]	0.027
**Age more than 70 years**	101 (59.4)	1 (14.3)	100 (61.3)	0.018
**Male sex**	108 (63.5)	5 (71.4)	103 (63.2)	0.996
**COVID-19 related**				
Previous COVID-19 dx before OHCA	3 (1.8)	0 (0)	3 (1.8)	0.999
Presumed symptoms before OHCA	20 (11.8)	1 (14.3)	19 (11.7)	0.591
chest PA after CPR	140 (82.4)	7 (100)	133 (81.6)	0.356
Abnormal chest x-ray findings	100 (58.8)	5 (71.4)	95 (58.3)	0.701
Acquired RT-PCR test	80 (47.1)	5 (71.4)	75 (46.0)	0.258
Final confirmation of COVID-19	10 (5.9)	0 (0)	10 (6.1)	0.999
**Comorbidities**				
Diabetes mellitus	52 (30.6)	2 (28.6)	50 (30.7)	0.999
Hypertension	59 (34.7)	3 (42.9)	56 (34.4)	0.695
Chronic renal disease	8 (4.7)	1 (14.3)	7 (4.3)	0.291
Malignancy, cancer	28 (16.5)	0 (0)	28 (17.2)	0.601
COPD, asthma	6 (3.5)	0 (0)	6 (3.7)	0.997
Heart failure, ischemic heart disease	26 (15.3)	0 (0)	26 (16.0)	0.597
Dementia, Parkinson disease	18 (10.6)	0 (0)	18 (11.0)	0.998
ICH, ischemic stroke	16 (9.4)	1 (14.3)	15 (9.2)	0.506
Liver cirrhosis, and others	17 (10.0)	0 (0)	17 (10.4)	0.998
**Location of OHCA, public place**	49 (28.8)	4 (57.1)	45 (27.6)	0.107
**Prehospital parameters**				
Witnessed event, anyone	129 (75.9)	6 (85.7)	123 (75.5)	0.994
Prehospital mechanical CPR	134 (78.8)	5 (71.4)	129 (79.1)	0.640
Prehospital defibrillation	22 (12.9)	3 (42.9)	19 (11.7)	0.047
**Time variables, median [IQR]**				
Response time interval (min)	8 [6–10]	8 [7–9]	8 [6–10]	0.743
Scene time interval (min)	21 [15–26]	15 [13–25]	22 [16–27]	0.205
**Prehospital ROSC before ED arrival**	8 (4.7)	6 (85.7)	2 (1.2)	< 0.001

*COPD* chronic obstructive pulmonary disease, *CPR* cardiopulmonary resuscitation, *ED* emergency department, *ICH* intracerebral hemorrhage, *IQR* interquartile ranges, *OHCA* out-of-hospital cardiac arrest, *ROSC* return of spontaneous circulation, *RT-PCR* reverse transcription polymerase chain reaction

### Factors associated with survival

**Fig. 2:**
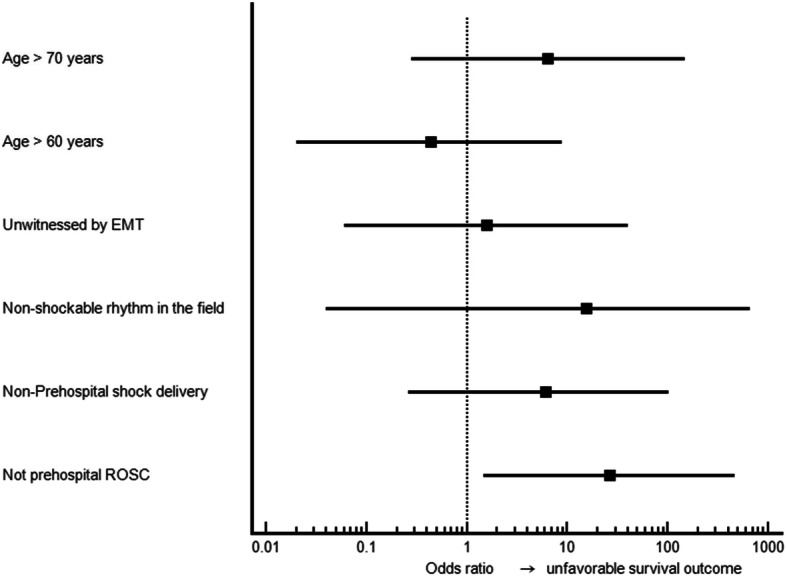
Forest plot for multivariate analysis of predictors of unfavorable survival of COVID-19 patients with OHCA. Data are adjusted for sex, age, location of event, witness status, bystander CPR, and any shockable rhythm

The rates of survival to discharge and survival with favorable neurological outcomes were 4.7 and 2.9%, respectively. Compared to the patients who died, the survivors were younger, experienced more prehospital defibrillations, and achieved ROSC before arriving at the ED (Table 2). Advanced age, arrest not being witnessed by emergency medical technician (EMT) personnel, receiving no shocks, and not developing ROSC before arriving at the ED were related to unfavorable survival outcomes. Only ROSC before arriving at the ED was related to survival to discharge (Fig. 2). Interestingly, age, witness status, and prehospital shock delivery were significantly associated with survival outcomes in the univariate analysis, but not in the multivariable analysis (Table 3).

**Table 3:** Logistic regressions of factors associated with unfavorable survival outcomes

	Dead	Survival	Univariate		Multivariable^a^	
			Crude Odds (95% CI)	***P*** value	Adjusted Odds (95% CI)	***P*** value
Age (years)	75 [63–80]	62 [57–69]	1.05 (1.00–1.10)	0.070		
Age > 70 years	100 (61.3)	1 (14.3)	9.52 (1.12–80.97)	0.039	6.43 (0.28–148.0)	0.244
Age > 60 years	127 (77.9)	4 (57.1)	2.64 (0.56–12.36)	0.216	0.44 (0.02–8.83)	0.591
Location, non-public places	118 (72.4)	3 (42.9)	3.49 (0.75–16.24)	0.107		
Not witnessed by bystander	64 (39.3)	3 (42.9)	0.86 (0.18–3.97)	0.999		
Not witnessed by EMT	139 (85.3)	4 (57.1)	4.34 (0.91–20.63)	0.081	1.56 (0.06–39.89)	0.782
No bystander CPR	108 (66.3)	4 (57.1)	1.47 (0.32–6.81)	0.691		
Initial acquired rhythm						
Non-shockable rhythm in the field	152 (93.3)	3 (42.9)	18.42 (3.65–92.8)	0.001	15.32 (0.04–663.7)	0.378
Asystole in the field	107 (65.6)	0 (0)	–	0.001	–	
Asystole in the ED	127 (77.9)	0 (0)	–	< 0.001	–	
No prehospital shock delivery	144 (88.3)	4 (57.1)	5.68 (1.18–27.4)	0.047	6.06 (0.26–101.1)	0.262
No prehospital ROSC	161 (98.8)	1 (14.3)	48.30 (3.83–109.3)	< 0.001	26.24 (1.48–463.3)	< 0.001

*CI* confidence interval, *CPR* cardiopulmonary resuscitation, *ED* emergency department, *EMT* emergency medical technician, *OR* odds ratio, *ROSC* return of spontaneous circulation

^a^Adjusted for age, gender, location of arrest, primary electrocardiogram (ECG), witness status, and whether prehospital ROSC was achieved before arriving at the ED

### External validation of the TOR rules

**Fig. 3:**
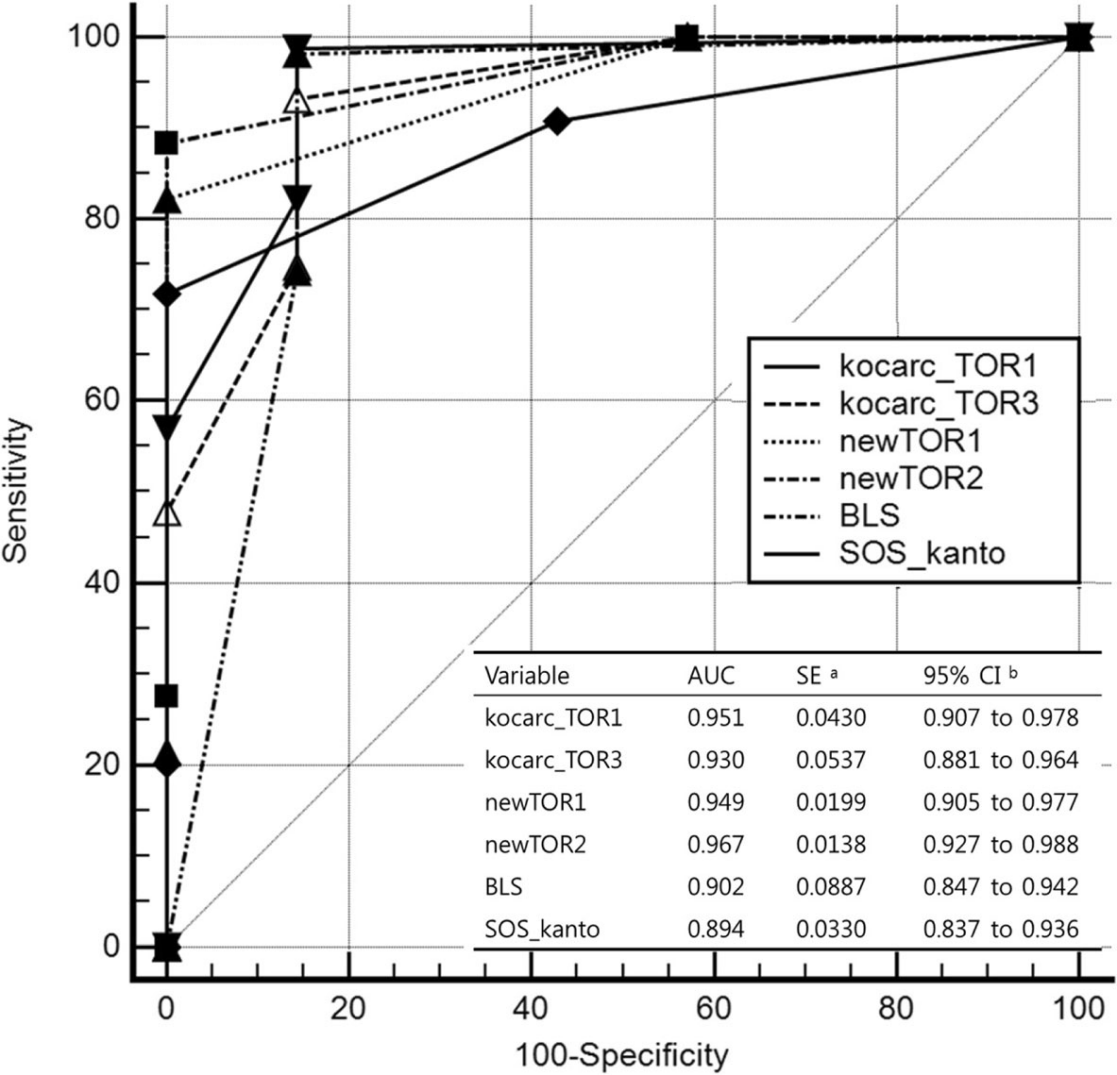
Receiver operating characteristic curves for BLS-TOR rules to predict poor survival outcomes at hospital discharge

On applying the existing international TOR rules to our registry, we found that 122 patients (71.8%) met all three criteria, but one survivor was discharged (Fig. 1). Another patient met the criteria for the traditional BLS-TOR and KoCARC TOR II rules, but he was also discharged (Table 4). The ROC curves of multimodal TOR categories for poor outcomes are shown in Fig. 2. Overall, KoCARC TOR rule I and the current TOR rule II, with AUCs of 0.951 and 0.967, respectively, were the most effective for predicting in-hospital mortality (Fig. 3). With respect to the specificity, FPR, FPV (narrow range of 95% CI) and PPV (> 99%), only KoCARC TOR rule I predicted poor survival outcomes, and it showed a higher diagnostic performance than did the other TOR rules (Table 4).

**Table 4:** External validations of multimodal TOR rules for predicting death prior to discharge (*n* = 170)

TOR rules		Death	Survival	Sensitivity (95% CI)	Specificity (95% CI)	FPV (95% CI)	PPV (95% CI)	NPV (95% CI)
**Before arriving at the ED**								
International BLS-TOR	met all criteria	121	1	74.2%	85.7%	0.8%	99.2%	12.5%
	did not fulfill	42	6	(66.7–80.6)	(42.0–99.2)	(0.04–5.2)	(84.8–99.9)	(5.2–25.9)
Goto’s rule	met all criteria	58	0	35.6%	100%	0%	100%	6.3%
	did not fulfill	105	7	(28.4–43.5)	(56.1–100)	(0–7.7)	(92.3–100)	(2.8–12.9)
KoCARC TOR rule I	met all criteria	93	0	57.1%	100%	0%	100%	9.1%
	did not fulfill	70	7	(49.1–64.7)	(56.1–100)	(0–4.9)	(95.1–100)	(4.0–18.4)
KoCARC TOR rule II	met all criteria	97	1	56.5%	85.7%	1.0%	98.9%	8.3%
	did not fulfill	66	6	(51.5–67.0)	(42.0–99.2)	(0.05–6.4)	(93.6–99.9)	(3.4–17.9)
KoCARC TOR rule III	met all criteria	78	0	47.9%	100%	0%	100%	7.6%
	did not fulfill	85	7	(40.0–55.8)	(56.1–100)	(0–5.8)	(94.2–100)	(3.4–15.6)
New TOR Model 1	met all criteria	35	0	21.5%	100%	0%	100%	5.2%
	did not fulfill	128	7	(15.6–28.7)	(56.1–100)	(0–12.3)	(87.7–100)	(2.3–10.8)
**After ED arrival**								
International ALS TOR	met all criteria	23	0	14.1%	100%	0	100%	4.8%
	did not fulfill	140	7	(9.3–20.6)	(56.1–100)	(0–17.8)	(82.2–100)	(2.1–9.9)
SOS-KANTO’s rule	met all criteria	33	0	20.2%	100%	0%	100%	5.2%
	did not fulfill	130	7	(14.5–27.4)	(56.1–100)	(0–12.9)	(87.0–100)	(2.3–10.6)
New TOR Model 2	met all criteria	45	0	27.6%	100%	0%	100%	5.6%
	did not fulfill	118	7	(21.0–35.2)	(56.1–100)	(0–9.7)	(90.2–100)	(2.4–11.6)

*ALS* advanced life support, *BLS-TOR* basic life support and termination of resuscitation, *CI* confidence interval, *ED* emergency department, *FPV* false positive value, *KoCARC* Korean Cardiac Arrest Research Consortium in 2015–2017; *NPV* negative predictive value, *PPV* positive predictive value, *SOS-KANTO* survey of survivors after cardiac arrest conducted in the Kanto area in 2012 (2017)

## Discussion

This study validated the existing multimodal TOR rules using the WinCOVID-19 Daegu registry data on OHCA patients. Among the nine existing TOR rules, KoCARC TOR rule I was found to be the best indicator of poor outcomes during the COVID-19 outbreak as evidenced by its highest specificity and PPV. To our best knowledge, this was the first study to determine the efficacy of these TOR guidelines for an emerging infectious disease.

Resuscitation of infected individuals greatly increases the risk of virus transmission to healthcare providers [1, 6, 16]. Current guidelines recommend emergency personnel to confirm the presence of COVID-19 in OHCA patients and wear high-level PPE, even in doubtful cases, before performing CPR. However, these measures may have limited benefits because COVID-19 has a very high rate of disease transmission and approximately 25% of the patients have asymptomatic infection [4]. Therefore, the COVID-19 era is a challenging and confusing time for healthcare workers in the medical and emergency fields [5]. Some OHCA patients were unexpectedly confirmed to be positive post-CPR or postmortem, contributing to emergency room shutdowns and temporary closures [17]. As of September 2020, more than 20,000 people have been confirmed to have COVID-19 in South Korea, and 385 of these patients have died. In addition, more than 50% of these cases were in Daegu, the area of interest in this study, and its neighboring area Gyeongbuk [10]. Further, more than 130 healthcare workers and medical staff have been infected. One physician died after treating two confirmed patients.

Our findings, although preliminary, showed that the survival outcomes of OHCA patients in Daegu during the peak of the COVID-19 outbreak (4.1%) were significantly lower than those reported nationwide (9.8%) or in the city (8.8%) in 2018 [18]. Many variables were changed in the COVID-19 era, including prehospital ROSC and pre-hospital transport time (see Additional file 1). Although we could not describe the impact of the COVID-19 pandemic on the chain of survival and its negative effects on high-quality CPR, the risk-benefit ratio for CPR should be reconsidered [2]. Despite that there were several factors associated with good prognosis in this study, including bystander CPR, EMS CPR, prehospital ROSC, and VF, no new factor was found for COVID-19. Other studies have also raised concerns on how CPR must be performed for IHCA patients with confirmed COVID-19. Considering the lower survival rate, physicians should establish goals of care or CPR preferences to reduce futile resuscitation by stratifying the survival probability of the IHCA patients, regardless of their COVID-19 status, at the time of hospital admission [5, 19]. It is also important to consider prehospital TOR for out-of-hospital resuscitation in an infectious disease epidemic area.

The previous TOR rules can be divided into two sets of variables: one can be applied at the pre-hospital level and the other can be evaluated immediately after arriving at the ED. In this study, we selected and analyzed the external validation of all nine multimodal TOR rules for OHCA patients during the COVID-19 epidemic period. These rules were commonly selected depending on the country or region where the derivation and validation phases were conducted. These included the (1) International BLS (a combination of three criteria: arrest not being witnessed by EMTs, not receiving prehospital shock delivery, and not experiencing prehospital ROSC, Table 1) and ALS rules derived and validated in the United States and Europe [8]; (2) Goto and KANTO-SOS rules developed in Japan and Asian countries [13, 14]; and (3) the Korean OHCA registry-based TOR models, KoCARC TOR rules, and two new TOR rules that were used in our previous studies [7, 11]. The international BLS-TOR rules that can be enforced at the prehospital stage has high sensitivity and specificity, but also relatively high FPR (1-specificity) [9, 15]. Therefore, a continuous development of the TOR model has been proposed [7, 9]. A previous study that included acquired ECG asystole rhythm as a criterion also proposed a new TOR model applicable at the prehospital stage and another TOR model applicable immediately after arriving at the ED [11].

The previous four rules have been partially validated in other countries and in the setting of mechanical CPR or comprehensive post-resuscitation care [12, 20–23]. Previous validations of the TOR rule reported survival rates of less than 1% among TOR rule–positive patients in North America. In contrast, high FPR of survival has been reported in Asian countries (28.7% in Singapore, 25.9% in Taiwan, and 30.4% in South Korea). This discrepancy may be due to different prehospital practices and a relatively higher prevalence of non-shockable rhythm in patients in Asian countries [7]. However, the high false positive cases of survival in these Asian countries, where the withdrawal of life-sustaining treatment is not commonly applied, are likely to be biased. Kajino et al. [24] validated the TOR rules for predicting poor neurologic outcomes in a Japanese population and concluded that more specific TOR rules for each region should be developed, despite the good performance of the TOR rules in their study. However, even if the COVID-19 outbreak was not considered, these previous results implied that the extrapolation to and implementation of different TOR rules in regions with different organization of EMS treatment protocols, legislation, and socioeconomic characteristics might be problematic because the TOR rules would need to be adjusted to meet the regional situation.

In this study, we validated the existing multimodal TOR rules using the WinCOVID-19 Daegu registry data on OHCA patients. Our results indicated that of the 170 OHCA patients, we failed to screen one survivor of the seven survival discharges for the international BLS and KoCARC II rules. However, the remaining seven TOR rules were classified correctly. Current guidelines recommend that diagnostic tests that guide the cessation of life-saving efforts be accurate and reliable, with an FPV and FPR value close to 0% [7, 9]. Among the nine rules, KoCARC TOR rule I was found to be the most effective indicator for poor outcomes, as indicated by the lowest FPV (0% with narrow 95% CI) and highest PPV (> 99%). This rule included the combination of three factors, namely, not being witnessed by EMT, presenting with an asystole at the scene, and experiencing no prehospital shock or ROSC. It did not include the patient’s age or ED parameters, thereby making it easy to use in prehospital settings and applicable for OHCA patients in this current pandemic [7].

This study had several strengths and limitations. First, as with other multicenter observational studies, the integrity of the data could be biased. In addition, the observation period was only 2 months; hence, the effects on long-term survival outcomes, which are most important for OHCA research, remain unknown. In this regard, we will continue to conduct investigations until the COVID-19 pandemic has been effectively contained. Second, it has been speculated that poor survival outcomes are associated with fewer resuscitations and prolonged EMS scene and transport time to the hospital. Moreover, unfavorable neurological outcomes, as a primary outcome, is more suitable than survival to discharge. Because only four patients in our study displayed favorable neurological outcomes, we could not perform any secondary analyses or external validations. Third, the ratio of the survival outcomes was markedly lower in our study than that in previous Korean OHCA reports during the COVID-19 outbreak. This could be due to the direct adverse effects of COVID-19 on the cardiovascular system, prolonged EMS scene time, and differences in treatment in different hospitals [25]. Fourth, the number of COVID-19 cases sharply decreased in April in Daegu. Thus, the sample size was too small for a large-scale multi-factor analysis. Finally, facility or regional differences in EMS resources, CPR quality, and post-cardiac arrest care might affect the survival outcomes during the COVID-19 outbreak. TOR rules in the COVID-19 era and socio-ethical issues must be discussed further, and a consensus process must be developed.

## Conclusions

Among the nine previously existing TOR rules, KoCARC TOR rule I is the most suitable for predicting poor survival outcomes, and it showed improved diagnostic performance in the COVID-19 era. With regard to the specificity, FPV and PPV criteria, KoCARC TOR rule I was superior to all other TOR rules. Further research on variations in resources and treatment protocols (CPR quality and post-cardiac arrest care) among facilities, regions, and cultures will be useful in determining the feasibility of TOR rules for COVID-19 patients worldwide.

## Supplementary Information

**Additional file 1.**

## Data Availability

The data supporting the findings of this study are available from the corresponding author upon reasonable request.

## Abbreviations

ALS: Advanced life support
AUC: area under the curve
BLS-TOR: Basic life support termination of resuscitation
CI: Confidence interval
COVID-19: Novel coronavirus disease
CPR: Cardiopulmonary resuscitation
ED: Emergency department
EMC: Emergency medical center
EMS: Emergency medical service
EMT: Emergency medical technician
FPV: False positive value
ICU: Intensive care unit
IHCA: In-hospital cardiac arrest
IQR: Interquartile range
KoCARC: Korean Cardiac Arrest Research Consortium
NPV: Negative predictive value
OHCA: Out-of-hospital cardiac arrest
OR: Odds ratio
PPE: Personal protective equipment
PPV: Positive predictive value
ROC: Receiver operating characteristic
ROSC: Return of spontaneous circulation
