# Supplementary material for: External validation of multimodal termination of resuscitation rules for out-of-hospital cardiac arrest patients in the COVID-19 era
Source: Scand J Trauma Resusc Emerg Med. 2021 Jan 27;29:19. doi: 10.1186/s13049-021-00834-0 (PMC7838848; doi:10.1186/s13049-021-00834-0)
Supplement: Supplementary file 1 — Additional file 1. [file 13049_2021_834_MOESM1_ESM.docx]

**Supplement 1.** Comparison of prehospital and hospital resources of out-of-hospital cardiac arrest before and after the COVID-19 outbreak

|  |  | non-traumatic , EMS-treated OHCA | | |  |
| --- | --- | --- | --- | --- | --- |
|  |  | 2017-2018 Nationwide, in South Korea | 2017-2018 Citywide, in Daegu | 2020.2.17-3.31 COVID-19 outbreak | p-value^a^ |
|  |  | N=44206 | N=2089 | N=170 |  |
| Basic epidemiology | male, sex | 27802 (62.8) | 1360 (65.1) | 108 (63.5) | 0.608 |
|  | Age, median [IQR] | 73.5 [60.2–80.3] | 74.3 [61.8–82.2] | 74.0 [62.0–80.0] | 0.589 |
|  | private or nursing home | 30363 (68.7) | 1487 (71.2) | 121 (71.2) | 0.907 |
|  | Any witnessed event | 23351 (52.8) | 1165 (55.7) | 129 (75.9) | <0.001 |
|  | Bystander CPR | 9892 (22.4) | 657 (31.4) | 58 (34.1) | <0.001 |
|  | prehospital VF/VT | 4351 (9.8) | 249 (11.9) | 15 (8.8) | 0.218 |
|  | Prehospital AED applied | 7461 (16.9) | 402 (19.2) | 22 (12.9) | 0.040 |
| EMS-related time interval (min) | Response time interval | 7 [5–10] | 6 [5–8] | 8 [6-10] | 0.009 |
|  | Scene time | 12 [8–18] | 13 [10–17] | 21 [15-26] | <0.001 |
| Prehospital CPR | Mechanical CPR | not reported | not reported | 134 (78.8) | - |
|  | Epinephrine, intravenous | not reported | 86 (4.1) | 63 (37.1) | <0.001 |
| Prehospital Airway | BVM, only others | not reported | 625 (29.9) | 61 (35.9) | 0.116 |
|  | SGA | not reported | 1,153 (55.2) | 89 (52.4) | 0.426 |
|  | Endotracheal intubation | not reported | 309 (14.8) | 16 (9.4) | 0.051 |
| Survival outcomes | Prehospital ROSC | 4951 (11.2) | 198 (9.5) | 8 (4.7) | 0.036 |
|  | Any survival, ROSC | 11567 (26.2)^b^ | 650 (31.1)^a^ | 38 (22.4) | 0.023 ^a^  0.318 ^b^ |
|  | Survival discharge | 4354 (9.8)^b^ | 183 (8.8)^a^ | 7 (4.1) | 0.065 ^a^  0.023 ^b^ |
|  | Favourable neurologic | 2803 (6.3)^b^ | 126 (6.0)^a^ | 4 (2.4) | 0.095 ^a^  0.067 ^b^ |

^a^*P* value: data analysis compared between 2017-2018 citywide and 2020 COVID-19 outbreak in Daegu, South Korea

^b^*P* value: data analysis compared between 2017-2018 nationwide and 2020 COVID-19 outbreak in Daegu, South Korea

Abbreviations: CPR, cardiopulmonary resuscitation; VF, ventricular fibrillation; VT, ventricular tachycardia; AED, automated external defibrillator; EMS, emergency medical services; BVM, bag-valve mask; SGA, supraglottic airway; ROSC, return of spontaneous circulation
